# The prevalence and incidence of delirium superimposed on dementia in community settings: A systematic review and meta‐analysis

**DOI:** 10.1002/dad2.70398

**Published:** 2026-06-18

**Authors:** Alice Burnand, Elizabeth L. Sampson, Kate Walters, Ayesha Dar, Jess Kay, Daniel Davis, Victoria Vickerstaff, Nathan Davies

**Affiliations:** ^1^ Research Department of Primary Care and Population Health, Centre for Ageing Population Studies University College London London UK; ^2^ Centre for Psychiatry and Mental Health, Wolfson Institute of Population Health QMUL London UK; ^3^ Institute of Health Informatics University College London London UK; ^4^ Centre for Evaluation and Methods Wolfson Institute of Population Health QMUL London UK

**Keywords:** community, delirium, delirium superimposed on dementia, dementia, incidence, occurrence, prevalence

## Abstract

Delirium superimposed on dementia (DSD) is a serious condition causing poor outcomes. There is a significant gap in understanding the epidemiology in community settings. This systematic review aimed to estimate the prevalence and incidence of delirium in community‐dwelling people with dementia. Medline, Embase, PsychINFO, Scopus, Web of Science, CINAHL, and Cochrane databases were searched from inception to January 6, 2025. Articles published in peer‐reviewed journals that focused on the prevalence, incidence, or occurrence of DSD were included. Twenty‐seven studies met the inclusion criteria. The overall pooled prevalence of DSD was 30.2%, with a sensitivity analysis of 27.7%. Prevalence varied by country, setting, and tool used for case ascertainment. Our prevalence findings align with previous research but indicate a higher‐than‐expected rate of DSD in community settings compared to what may be seen in practice. Future research should address the heterogeneity and determine the validity of screening tools.

## Highlights

- Twenty‐seven studies met the inclusion criteria.
- Prevalence of delirium superimposed on dementia (DSD) in the community varies considerably.
- Pooled prevalence of DSD was 30.2%.
- Pooled incidence was 79.0 cases per 1000 person‐years.

## 1 INTRODUCTION

Delirium and dementia commonly coexist, known as “delirium superimposed on dementia” (DSD).^1^ Delirium is a medical emergency that impairs neurological function with a breakdown of neuronal connectivity and inflammation.^2^ It is characterised by fluctuations in cognition, memory, and attention, as well as behavioral changes such as anxiety, hallucinations, or withdrawal, in which onset is acute.^1^ In contrast, dementia is a clinical syndrome of progressive cognitive decline caused by neurodegenerative, vascular, or mixed diseases.^3^ The type of dementia will influence the course and clinical presentation.^4^ Dementia is a risk factor most strongly associated with developing delirium.^4^ There are many negative consequences of DSD for both patients and family caregivers, particularly if unrecognised and left untreated, which include greater irreversible cognitive and functional decline, longer hospital stays, distress, burden, anxiety, nursing home admission, and mortality.^5^, ^6^

Distinguishing between these two separate conditions or diagnosing their coexistence in a patient can be challenging, particularly with persistent or recurrent delirium, or rapidly progressing dementia.^1^ The clinical presentations of different dementia subtypes often overlap with delirium symptoms, complicating accurate diagnosis and frequently lead to under‐detection.^7^, ^8^ For instance, memory loss and impairment of daily functioning is more commonly seen in early stages of Alzheimer's disease (AD) and vascular dementia^9^ while hallucinations and delusions occur more frequently in Lewy body and Parkinson's disease dementia, affecting up to 80% of individuals with these diagnoses.^10^

Delirium has clinical subtypes based on psychomotor disturbance. The hyperactive form is often characterised by agitation, delusions, hallucinations, and disorientation, whereas hypoactive is characterised by confusion and sedation.^11^ People with the hypoactive type may also experience psychosis but it is difficult to assess their mental state to identify hallucinations or delusions. Furthermore, a related subthreshold state to delirium is subsyndromal delirium (SSD) which is reported as an intermediate stage between delirium and normal mental status but lacks standardised definition even in the Diagnostic and Statistical Manual of Mental Disorders (DSM)‐5.^12^ Understanding these nuanced differences between subtypes of dementia and delirium is important to establish accurate prevalence rates of DSD and its epidemiological burden.

While research on delirium prevalence exists within hospital contexts and the general population, there remains a notable gap in understanding DSD, especially its presentation in community‐dwelling individuals. There is also a lack of reporting of incidence of DSD in the community.

The prevalence of delirium in the community is generally low, estimated at 1% to 2%.^13^ However, among individuals aged ≥ 85 years the period prevalence over a 3‐year follow‐up has been reported to be 10%.^14^ In long‐term care facilities, delirium point and period prevalence range considerably, with reports of prevalence rates of 6.5% of delirium and 39.7% of SSD (period prevalence over 2 weeks),^15^ 17.5% (period prevalence over 28 days)^16^ and a high point prevalence estimation of 39%.^14^ Moreover, a study comprising nursing home residents (50% of the sample with diagnosis of dementia) report an incidence of 0.85 episodes of delirium per 100 person‐weeks and a period prevalence of 33%.^17^

Notably, the reported prevalence of DSD is considerably higher than that of delirium in the general population, ranging between 22% and 89% in a systematic review conducted more than two decades ago, which included both community and hospital settings.^18^ More recently, a meta‐analysis focusing on hospital settings reported a pooled DSD prevalence of 49%.^6^ However, to date, no comprehensive review has specifically estimated the pooled prevalence of DSD exclusively for people residing in community settings, such as in their own homes, nursing homes, or residential homes. Therefore, this systematic review and meta‐analysis aimed to understand the prevalence and incidence of DSD in community settings.

## 2 METHODS

This review was registered with PROSPERO (CRD42025645326) and Open Science Framework^19^ and is reported in accordance with the PRISMA (Preferred Reporting Items for Systematic Reviews and Meta‐Analyses) statement^20^ (Appendix S1 in supporting information).

### 2.1 Search strategy

We initially conducted a Medline pilot search to identify DSD prevalence and incidence medical subject heading (MeSH) terms and keywords, refined with literature review and an information specialist from University College London (UCL) library. A systematic search was then conducted across Medline, Embase, PsychINFO, Scopus, Web of Science, CINAHL, and Cochrane databases, from inception to January 6, 2025. The search combined keywords and subject headings for “dementia,” “confusion,” “delirium,” AND “community,” and associated synonyms (Appendix S2 in supporting information). Additionally, we conducted forward and backward citation tracking of included articles and contacted authors directly to obtain any unavailable full texts.

### 2.2 Eligibility criteria

Studies were eligible if:

1. They included participants with a diagnosis of any type of dementia.
2. Validated methods were used for identifying delirium, for example, the confusion attention method (CAM).^21^
3. They reported the prevalence or incidence of delirium and dementia.
4. The participants lived in community settings.

Studies were excluded if:

1. They had mixed sample and analysis of people with dementia with mild cognitive impairment or no cognitive impairment.
2. Validated tools were not used to identify delirium.
3. They did not report prevalence or incidence rates.
4. Participants were based in hospital settings or the paper stated they had been discharged from the hospital to be cared for at home.

The full inclusion and exclusion criteria are reported in Appendix S3 in supporting information.

All identified articles were exported to Rayyan^22^ for deduplication. After this, title and abstract screening were performed in duplicate by three reviewers (A.B.: 100%; A.D. and J.K.: 50% each). This was followed by full‐text assessment, also conducted in duplicate by the same reviewers (A.B.: 100%; A.D. and J.K.: 50% each), against the inclusion and exclusion criteria. Disagreements were resolved through discussion with the wider research team. Study authors were contacted for additional information or clarification to aid the screening and decision process when required.

### 2.3 Data extraction

Using a standardised data extraction form in Microsoft Excel, three reviewers independently extracted information. A.B. extracted data from all included papers, while A.D. and J.K. each extracted data from 50% of the papers. The extracted information included: authors, publication year, country of publication, study aim and objectives, study design, participant details (age, sex, place of residence, stage/type of dementia), and diagnostic/screening tools for both dementia and delirium, as well as reported prevalence and/or incidence of DSD (Appendix S4 in supporting information).

### 2.4 Quality assessment

Quality assessment was conducted independently by three reviewers (A.B.: 100% of papers; A.D. and J.K.: 50% each) using the Joanna Briggs Institute (JBI) critical appraisal checklists^23^ (Appendix S5 in supporting information). Different checklists for each study design were used, which included checklists for prevalence studies, cross‐sectional, cohort, randomised controlled trial (RCT), and case–control studies, and contained a variety of risk of bias areas. Any discrepancies in quality ratings were resolved through discussion among the three reviewers (A.B., A.D., and J.K.), with referral to the wider research team.

### 2.5 Statistical analysis

Meta‐analyses were performed using IBM SPSS Statistics, version 22.0^24^ and a random effects model was used for all meta‐analyses to account for anticipated heterogeneity. Prevalence was calculated by dividing the number of people with DSD by the total population of people with dementia. For both prevalence and incidence meta‐analyses, standard deviation, standard error, and confidence intervals were calculated for all proportions. Sensitivity analyses excluding papers with four or more potential areas of risk of bias were conducted. To present the extent of the between‐study variation, prediction intervals were calculated for the main pooled analysis and sensitivity analysis. Subgroup analyses for the prevalence meta‐analyses were conducted based on: (1) country of study, (2) the tool for delirium identification, and (3) the study setting. For the incidence meta‐analysis, raw incidence data were further converted to cases per 1000 person‐years to ensure a standardised rate for synthesis. These incidence rates were then log‐transformed for the meta‐analysis and subsequently back‐transformed to facilitate interpretation of the pooled results.

## 3 RESULTS

**FIGURE 1:**
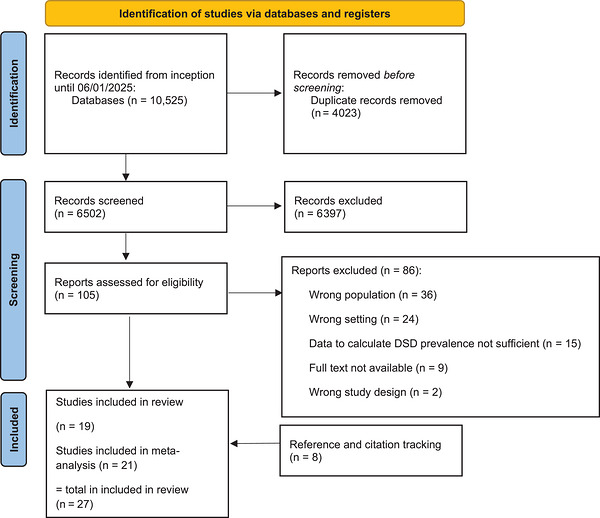
PRISMA flow diagram of the screening process. DSD, delirium superimposed on dementia; PRISMA, Preferred Reporting Items for Systematic Reviews and Meta‐Analyses.

The initial search identified 10,525 potential citations. After deduplication, 6502 titles and abstracts were independently dual screened. Subsequently, 105 articles underwent full‐text review against the eligibility criteria, leading to the exclusion of 86 studies. Reference and citation tracking identified an additional 8 eligible papers. In total, 27 papers were included in this systematic review, and 21 papers were eligible for meta‐analysis. Figure 1 displays the PRISMA flow diagram.

### 3.1 Study characteristics

Participants across studies lived in community settings, including nursing homes/residential care (*n* = 13), at home (*n* = 3), mixed home and residential care settings (*n* = 3), or were classified as community dwelling (*n* = 8). Study designs varied, including retrospective cohort (*n* = 5), prospective cohort (*n* = 8), cross‐sectional (*n* = 7), RCT (*n* = 4), and case–control (*n* = 3). Most (*n* = 24) were in Western Europe, with one from Japan^25^ and two from Turkey.^26^, ^27^ Sample sizes ranged from 34^28^ to 9324,^29^ with all participants aged ≥ 65 years. Dementia subtypes were inconsistently reported; when specified (*n* = 12), populations included only AD (*n* = 4) or mixed subtypes (*n* = 8). Dementia severity, also inconsistently reported, ranged from mild to severe. Delirium subtypes were not reported in any studies. See Appendix S6 in supporting information for full characteristics.

Dementia diagnoses used DSM‐III/IV/V criteria,^30^ Alzheimer's Disease Assessment Scale Cognitive subscale,^31^ Informant Questionnaire on Cognitive Decline in the Elderly,^32^ medical records, or International Classification of Diseases 10th Revision codes. Delirium was diagnosed/screened via DSM‐III/IV/5 criteria,^30^ CAM,^21^ 4‐Assessment Test (4AT),^33^ or observational assessment/clinical judgment. See Table 1 for reported prevalence and Table 2 for incidence.

**TABLE 1:** Prevalence of DSD in studies focusing on delirium or delirium superimposed on dementia.

Study	How dementia was diagnosed	How delirium was diagnosed/screened	Who diagnosed delirium	Total participants (*n*) with dementia	Total participants with DSD (*n*)	Prevalence of DSD (%) with 95% CIs	Point or period prevalence
**Study setting: nursing home/residential care**							
Bogaerts et al. (2024)^34^	Reisberg Global Deterioration Scale	CAM short	Nurse	205	Probable delirium: 36 delirium: 21 total: 57	Probable delirium: 17.56% delirium: 10.24% total: 27.8%, (95% CI 22%–34%)	Point prevalence
Boorsma et al.^35^	Not reported	NH‐CAM	Nurses	771	110	14.27% (95% CI 11.80%–16.73%)	Point prevalence
Eriksson et al. (2008)^36^	DSM‐IV	Questionnaire about episodes of delirium in the past month	Nurse who knew the residents well	103	35	33.98% (95% CI 24.83%–43.13%)	Point prevalence
Katipoglu et al.^27^	DSM‐IV and DSM‐V	CAM and DSM‐IV & DSM‐V	Geriatricians	721	195	27.05% (95% CI 23.80%–30.29%)	Period prevalence
McCusker et al.^37^	Extracted from medical charts	CAM	Research assistant supervised by a study psychiatrist	Cohort B: MMSE score ≤10: AD: 25 VD: 3 Mixed: 4 Others & not specific: 19 Total with dementia: 51 Without dementia: 4 Total *n* = 55	16	Total (*n* = 55): 29.1% (95% CI 17.88%–41.09%)	Point prevalence
Mentes et al.^38^	Not reported	One the five delirium indicators: (1) less alert/easily distracted, (2) changing awareness of environment, (3) episodes of incoherent speech, (4) periods of motor restlessness or lethargy, (5) cognitive ability variations over the day, which were present over the previous days, and were different from the resident's usual functioning	Not reported	1027	170	16.55% (95% CI 14.28%–18.82%)	Point prevalence
Morichi et al.^39^	Not reported	4AT	Mainly geriatricians with long‐term work experience in NHs	754	401	53.18% (95% CI 49.62%–56.74%)	Point prevalence
Oudewortel et al. (2021)^40^	All records on baseline with AD or dementia other than AD were used.	A diagnosis of delirium was based on the criteria recorded in the SHELTER database: (1) an acute change in mental status deviating from usual functioning within the 3 days before the assessment OR a new onset or worsening of 1 or more of the following symptoms: (1) easily distracted, (2) episodes of disorganised speech, (3) mental function variation over the course of the day	Most assessors were nurses	2108	444	21.06% (95% CI 19.32%–22.80%)	Point prevalence
Sandberg et al. (1998)^41^	DSM‐III‐R	DSM‐III‐R	A “trained personnel with considerable psychiatric training and professional experience.”	Study participants not all of whom had dementia: Nursing homes *n* = 202 “Old people's homes” *n* = 196 Home medical care *n* = 171	Not reported raw scores of DSD, only %.	Nursing home: 42.1% Old people's homes”: 8.2% Home medical care: 9.9% Unable to calculate CIs.	Point prevalence
Santagata et al. (2021)^42^	Not reported	CAM	Not reported	52	15	28.84% (95% CI 16.53%–41.17%)	Point prevalence
Skretteberg et al. (2022)^43^	Medical records	CAM	The nurse, health‐care worker, or physician who best knew the patient. The CAM was performed by 2 of the staff	116	28	24.13% (95% CI 16.35%–31.93%)	Point prevalence
Zazzara et al. (2022)^44^	Identified via the assessment instrument interRAI Long‐Term Care Facility.	An episode of delirium was defined by the presence of at least one of the following three symptoms in the 3 days prior the interview: (1) abnormal thought process, (2) delusions, (3) hallucinations	Health professional assessor	2563	833	32.50% (95% CI 30.69%–34.31%)	Point prevalence
**Study setting: community dwelling**							
Fick et al.^29^	Using ICD‐9‐CM codes	Using ICD‐9‐CM codes	Not reported	7347	976	13.28% (95% CI 12.51%–14.06%)	Point prevalence
Holmes et al. (2011)^45^	NINCDS‐ADRDA criteria for probable or possible AD	CAM	Caregivers	300	25	8.33% (95% CI 5.21%–11.46%)	Period prevalence
Manni et al. (2020)^46^	DSM‐V	CAM	Trained geriatrician	2995	109	3.64% (95% CI 2.97%–4.31%)	Point prevalence
Mathillas et al. (2013)^47^	DSM‐IV	DSM‐IV	Geriatrics specialist	299	156	52.17% (95% CI 46.51%‐57.83%)	Point prevalence
Quispel‐Aggenbach et al. (2021)^28^	DSM‐IV‐TR	DSM‐IV‐TR	A geriatrician and a registered psychiatric nurse	34	Possible delirium: 3 Probable delirium: 9 total: 12	Possible delirium: 8.82% Probable delirium: 26.47% Total: 35.29% (95% CI 19.23%–51.36%)	Point prevalence
Tremolizzo et al.^48^	Not reported	4AT	GP A geriatrician and a registered psychiatric nurse	35	32	91.43% (95% CI 82.15%–100%)	Point prevalence
**Study setting: outpatient department or hospital**							
Hasegawa et al. (2010)^25^	DSM‐III‐R	DSM‐III‐R	Senior neuropsychiatrists	206	40	19.42% (95% CI 14.02%–24.82%)	Point prevalence
Katipoglu and Naharci^26^	DSM‐IV & DSM‐V	DSM‐IV	Geriatrician	615	170	27.64% (95% CI 24.12%–31.18%)	Point prevalence
Stroomer‐van Wijk et al. (2016)^49^	DSM‐IV	DMS‐IV	An experienced psychogeriatrician	68	24	35.29% (95% CI 23.94%–46.65%)	Point prevalence
Vida et al. (2006)^50^	The presence and severity of dementia was ascertained using the short form of the IQCODE	CAM	Interviewer assessment and proxy report	132	60	45.45% (95% CI 36.96%–53.95%)	Point prevalence

Abbreviations: 4AT, 4‐Assessment Test; AD, Alzheimer's disease; CAM, Confusion Assessment Method; CI, confidence interval; DSD, delirium superimposed on dementia; DSM, Diagnostic and Statistical Manual of Mental Disorders; GP, general practitioner; ICD‐CM, International Classification of Diseases, clinical modification; IQCODE, Informant Questionnaire on Cognitive Decline in the Elderly; MMSE, Mini‐Mental State Examination; NH, nursing home; NINCDS‐ADRA, National Institute of Neurological and Communicative Disorders and Stroke and Alzheimer's Disease and Related Disorders Association Criteria for Alzheimer's Disease; TR, text revision; VD, vascular dementia.

**TABLE 2:** Incidence of delirium in dementia in studies focusing on delirium‐superimposed on dementia.

Study	How dementia was diagnosed	How delirium was diagnosed	Who diagnosed delirium	DSD incidence
**Study setting: long‐term care**				
Cole et al.^51^	Not reported	[report SSD] CAM SSD1: required the presence of one or more new CAM core symptoms that did not meet CAM criteria for delirium and did not progress to delirium.	Nurses and psychologists with experience in geriatrics and discussion with clinical investigator.	Time at Risk (SSD1): 687 person‐weeks (*n* = 33) Sensitivity analysis*: Time at Risk (SSD1): 557 person weeks (*n* = 30) AD: Time at Risk (SSD1): 185 person‐weeks (*n* = 6) VD: Time at Risk (SSD1): 90 person‐weeks (*n* = 7) Other and not specified: Time at Risk (SSD1): 196 person weeks (*n* = 12) Mixed: Time at Risk (SSD1): 86 person‐weeks (*n* = 5) * the sensitivity analyses excluded episodes of SSD1 that continued to the end of the 6‐month period of observation, as these may have progressed to delirium and been misclassified as episodes of SSD. Incidence rates: Incidence rate: 4.8 cases of SSD1 in people with dementia per 100 person‐weeks. Sensitivity analysis**: Incidence rate: 5.4 cases of SSD1 in people with dementia: per 100 person‐weeks. AD: 3.2 cases of SSD1 in people with dementia: per 100 person‐weeks. VD: 7.8 cases of SSD1 in people with dementia: per 100 person‐weeks. Other and not specified: 6.1 cases of SSD1 in people with dementia: per 100 person‐weeks. Mixed: 5.8 cases of SSD1 in people with dementia: per 100 person‐weeks.
McCusker et al.^37^	Extracted from medical charts	CAM	Research assistant supervised by a study psychiatrist	Incidence rate: 6.9 cases of DSD per 100 person‐weeks.
**Study setting: general practice**				
Delgado et al. (2022)^52^	Medical records from primary and secondary care.	Incidence of adverse health outcomes recorded in primary care records.	Not reported	Incidence rate: 53.7 cases of DSD per 1000 person‐years For meta‐analysis: log incidence rate = 3.98 (SE 0.05, 95% CI 3.88–4.08)
**Study setting: community**				
Dyer et al. (2020a)^53^	NINCDS‐ADRDA criteria	Family CAM	Family carers in interviews	11.38% experienced incident delirium within 18 months of the study period. *n* = 21/51 (41.18%) of these experienced > 1 episode of delirium within 18 months. Incidence rate: 80.5 cases of DSD per 1000 ‐person‐years For meta‐analysis: log incidence rate = 4.39 (SE 0.14; 95% CI 4.12–4.66)
Dyer et al. (2020b)^54^	NINCDS‐ADRDA criteria	Family CAM	Caregivers	11.17% experienced incident delirium within 18 months of the study period. Incidence rate: 79.0 cases of DSD per 1000 person‐years (SE 0.13, 95% CI 4.11–4.62) For meta‐analysis: log incidence rate = 4.37
Lerner et al. (1997)^55^	NINCDS‐ADRDA criteria	Presence of delirium defined by: (1) a sudden behavioral change from baseline; (2) increased or decreased psychomotor activity; (3) increased confusion, agitation, new onset of sleep–wake cycle disturbance lasting over 24 hours. In accordance with DSM III‐R, all cases of delirium had a recognisable etiologic factor.	Information obtained from primary caregiver by a physician and medical records	Incidence of DSD was 21.61% within 3 years of follow‐up in the study Incidence rate: 81.2 cases of DSD per 1000 person‐years For meta‐analysis: log incidence rate = 4.40 (SE 0.15, 95% CI 4.11–4.67)

Abbreviations: AD, Alzheimer's disease; CAM, Confusion Assessment Method; CI, confidence interval; CSDD, Cornell Scale for Depression in Dementia; DI, delirium index; DSM, Diagnostic and Statistical Manual of Mental Disorders; HDS, Hierarchic Dementia Scale; HR, hazard ratio; MMSE, Mini‐Mental State Examination; NINCDS‐ADRDA, National Institute of Neurological and Communicative Disorders and Stroke/Alzheimer's Disease Criteria; SE, standard error; SSD, subsyndromal delirium; VD, vascular dementia.

### 3.2 Study quality

Most studies were rated to have none or one potential area of risk of bias (*n* = 16), but some (*n* = 9) had two to three areas of potential risk of bias; these were mainly cohort studies (*n* = 6) in which confounding variables were not stated, follow‐up was not complete, nor were strategies for incomplete follow‐up conducted. There were two studies that were rated with four or more areas of potential risk of bias, which varied across addressing confounding variables, reporting the inclusion criteria, and not reporting the details of the sample population or the sample size justification (Appendix S5).

### 3.3 Delirium prevalence

**FIGURE 2:**
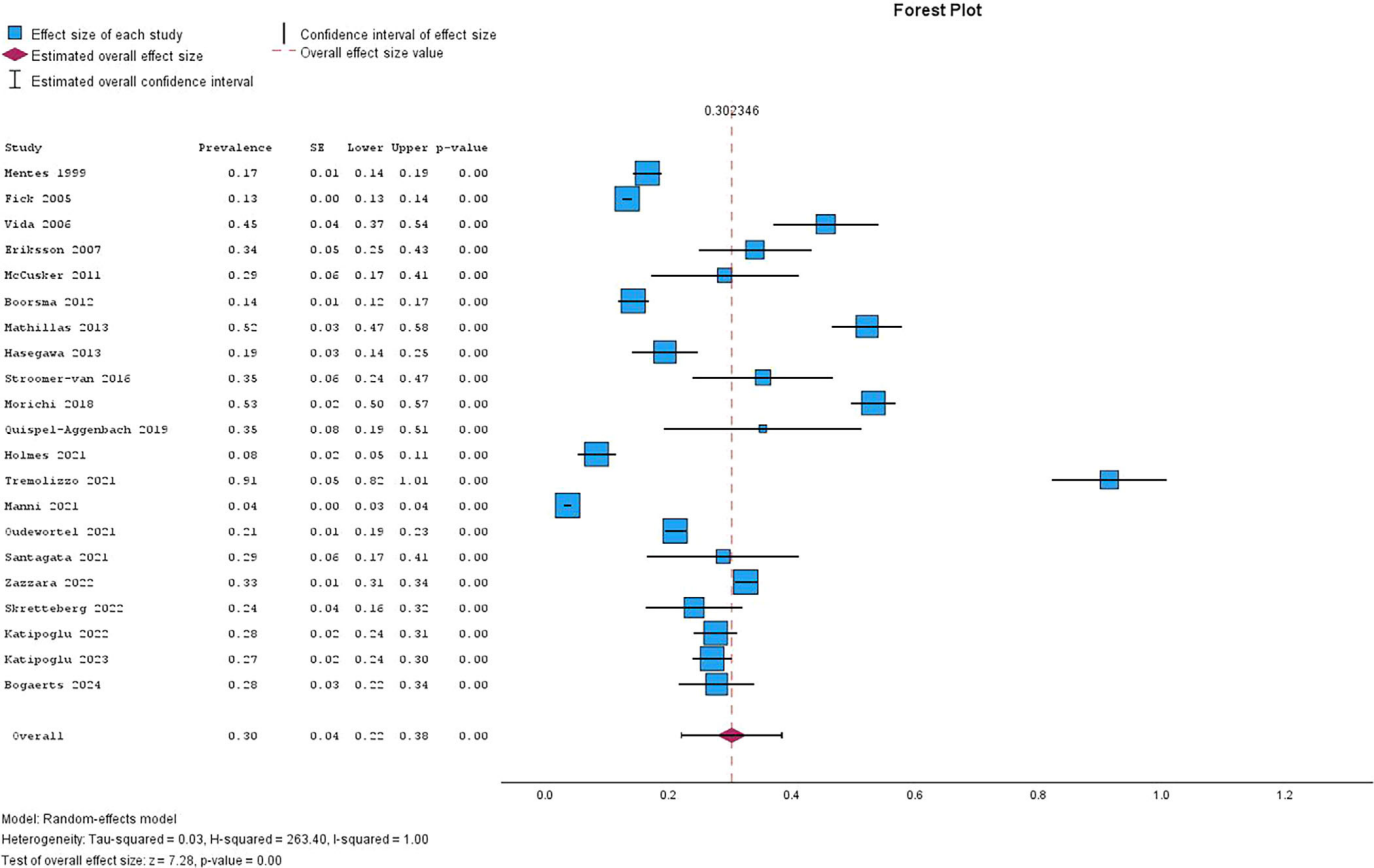
Forest plot of total pooled prevalence.

A meta‐analysis (including *n* = 21 studies) estimated the pooled prevalence of DSD at 30.2% (95% confidence interval [CI]: 22.1%–38.4%), stratified by reference and ordered by year (Figure 2). This estimate was accompanied by high heterogeneity, indicated by an *I* ^2^ value of 1.00 and a *p* value of < 0.01 (Figure 2, Appendix S7A in supporting information). The prediction intervals (PIs) were calculated at 95% PI: 0% to 61%. A sensitivity analysis was conducted, excluding two papers^38^, ^48^ that contained four or more areas of potential risk of bias. This analysis yielded a pooled prevalence estimate of 27.7% (95% CI: 21.4%–33.9%; 95% PI: 0%–58.1%; Appendix S7B and S7C).

To understand regional variation, a subgroup meta‐analysis was conducted across different geographical regions, including all eligible papers (*n* = 21; Appendix S7D). The pooled prevalence for European countries was estimated at 32.1% (95% CI: 22.0%–42.2%), while the pooled prevalence for the rest of the world was lower, at 24.2% (95% CI: 12.9%–35.5%). However, Tremolizzo et al.,^48^ which displayed an estimated effect size of 0.91, which is considerably higher than the other studies in both the Europe and rest of the world subgroup, presents as an outlier and is likely to impact this pooled estimate. Excluding Tremolizzo et al.^48^ and an additional paper^38^ with four areas of potential risk of bias reduced the Europe pooled prevalence to 28.1% (95% CI: 20.9%–35.2%) and marginally increased the rest of the world's prevalence to 26.3% (95% CI: 12.4%–40.3%; Appendix S7E).

All participants in the included studies lived in community settings; however, the study site location where data collection and recruitment occurred varied among “community dwelling,” nursing homes, and hospital outpatient clinic settings (participant attended outpatient services for the study while living at home). A subgroup meta‐analysis (Appendix S7F) of these three settings indicated a pooled prevalence of 33.8% (95% CI: 6.9%–60.7%) for community‐dwelling individuals, 27.9% (95% CI: 21.4%–34.4%) for those living in nursing homes, and 31.4% (95% CI: 20.5%–42.4%) for studies conducted in hospital outpatient clinic settings. Excluding papers with four or more areas of risk^38^, ^48^ in a sensitivity analysis reduced community prevalence to 22.1% (95% CI: 3.9%–40.2%) and increased nursing home pooled prevalence to 29.2% (95% CI: 22.4%–35.9%; Appendix S7G).

Subgroup analyses (Appendix S7H) based on delirium diagnostic and screening tools revealed pooled prevalence estimates of 22.1% (95% CI: 12.6%–31.6%) for the CAM; 32.5% (95% CI: 22.9%–42.1%) for the DSM‐III, IV, or 5; and 24.8% (95% CI: 15.1%–34.4%) for delirium identified by predetermined criteria and clinical judgment. The two studies utilising the 4AT^39^, ^48^ were not included in this subgroup meta‐analysis but reported high prevalences of 53.2% (95% CI: 50%–57%) and 91.4% (95% CI: 82%–100%).

### 3.4 DSD incidence

**FIGURE 3:**
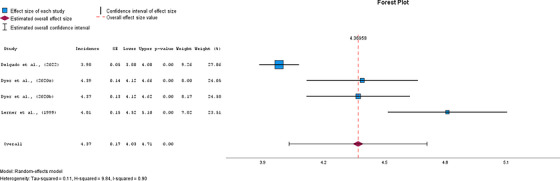
Forest plot of log incidence rates per 1000‐person years.

Incidence rates are reported as cases per 1000 person‐years for the four eligible studies. The pooled incidence rate was determined to be 79.0 cases per 1000 person‐years (95% CI: 56.3–111.1) after back‐transformation for interpretation (Figure 3; Appendix S7H). For the studies not included in the meta‐analysis, Cole et al.^51^ reported an incidence of SSD of 4.8 per 100 person‐weeks among participants with dementia (*n* = 33). A sensitivity analysis in the same study, excluding episodes of SSD that continued past the 6 months of observation, indicated a slightly higher incidence of 5.4 per 100 person‐weeks when considering a subset of 30 participants. Separately, McCusker et al.^37^ found a DSD incidence of 6.9 per 100 person‐weeks (*n* = 55).

To allow comparisons to the literature included in this review, which report incidence as 100 person‐weeks, this incidence was converted. Incidence of 79.04 cases per 1000 person‐years is equivalent to an incidence of 0.15 per 100 person‐weeks.

Moreover, to further convert this to a percentage, to compare across literature when incidence is reported as a percentage over a year (cumulative incidence), 79.04 cases per 1000 person‐years was converted to 7.6% full‐year (cumulative) incidence.

All incidence results are detailed in Table 2.

## 4 DISCUSSION

To our knowledge, this is the first review to systematically estimate the prevalence and incidence of DSD in community settings. Our sensitivity analysis show that delirium is prevalent in 27.7% of community‐dwelling people with dementia aged ≥ 65. Subgroup analyses indicate differences in prevalence across various settings: 22.1% in community‐dwelling individuals (sensitivity analysis), 29.2% in nursing homes (sensitivity analysis), and 31.4% in outpatient hospital settings. Prevalence also varied based on the method used for delirium identification: 22.1% with the CAM, 32.5% with DSM criteria, and 24.8% with clinical judgment. On average, there were 79.0 incident cases of DSD for every 1000 person‐years of observation. All pooled analyses demonstrated a high level of heterogeneity (prevalence *I* ^2 ^= 1.0, *P* < 0.01; incidence *I* ^2 ^= 0.90, *p* = 0.31). Moreover, the wide‐ranging prediction interval (0%–59%) indicates a high level of variability and uncertainty about the true prevalence of DSD. However, such variations in prevalence and incidence estimates are expected given differences in study design, setting, sample characteristics, case ascertainment, and definitions.

Reported prevalence and incidence of delirium in different settings varied considerably. The lowest reported prevalence stands at 1.4% of people in long‐term care with a sample including 60% who were “cognitively impaired.”^56^ For populations exclusively with dementia this prevalence is higher, with a reported prevalence of DSD in hospital settings at 48.9%^6^ and in mix of long‐term care facilities and hospital was found to be as high as 70.3%.^57^ A study in 2019 reported people with Lewy body dementia (LBD) had a higher incidence of delirium (6.2 per 100 person‐years) than people with AD (2.3 per 100 person‐years).^58^ Moreover, a study of delirium incidence in nursing homes in a sample containing ≈ 50% with dementia found a higher rate of 20.7 per 100 person‐years and in residential homes 14.6 per 100 person‐years.^35^

Our prevalence findings align with previous research, yet they indicate a higher‐than‐expected rate of DSD in community settings compared to what may be seen in clinical practice. This suggests a notable discrepancy between research and clinical findings. The tools used for screening and case ascertainment may explain this heterogeneity. Research on the performance of delirium screening tools in people with dementia in the community is limited and inconclusive.^59^ The 4AT was designed for screening in acute settings and the CAM for diagnosis; both are validated for use in hospitals.^21^, ^33^ Therefore, these tools may not be fully reliable in identifying DSD in the community and may need further refinement and validation.

Furthermore, within the research included in our meta‐analysis, expertise of the individuals applying the screening tool varied. The CAM was delivered by a wide range of individuals, including carers, research assistants, health‐care workers, and nurses, whereas the studies that diagnosed delirium guided by the DSM were primarily administered by experienced geriatricians. The CAM has reduced sensitivity when applied by people who are not trained^59^ and therefore less experienced staff may have found it more challenging to confidently differentiate symptoms of delirium from those of dementia. Furthermore, studies utilising the DSM predominantly used the less restrictive DSM‐IV compared to the DSM‐5^60^ due to the availability of the DSM version at the time of the study and therefore the higher sensitivity may lead to increased cases of identification due to false positives.

A sensitivity analysis excluding papers with four or more areas of risk of bias (Mentes et al.^38^); (Tremolizzo et al.^48^) reduced the initial pooled DSD prevalence observed in community‐dwelling individuals’ prevalence from 33.8% to 22.1%. This adjustment excluded a sample of housebound participants, who had programmed visits from general practitioners due to their frailty and vulnerability, and likely possessed a higher prevalence of chronic conditions. Thus, the sample resembled frail individuals in residential care with a potentially higher risk of delirium and inflated the initial community‐dwelling prevalence. This also suggests the term “community dwelling” is heterogeneous and can include people with dementia with different health profiles.

The varying prevalence may also be due to the different subtypes and severity of dementia. In the early stages of dementia, DSD may be easier to identify, with a greater dependency on carers’ ability to identify changes suggestive of delirium in advanced stages.^7^ As severity and subtype of dementia in the included studies were seldom consistently reported, it is difficult to draw conclusions about whether the heterogeneity of prevalence reported is due to the inability to accurately identify delirium in the varying populations with dementia.

This review has some limitations. Foremost, there is observed asymmetry in the funnel plots (Appendix S7A–S7F), which suggests the possibility of publication bias or small study effects. While excluding high‐risk papers in sensitivity analysis showed a slight improvement, some asymmetry persisted. Most studies were conducted in Western contexts primarily the United States or with European populations, potentially limiting the generalisability of our results to other settings, in particular low‐income and middle‐income countries.

In conclusion, this meta‐analysis suggests delirium affects 27.7% of community‐dwelling older adults with dementia and reports an incidence of 79.0 cases per 1000 person‐years. Noteworthy findings include a clear discrepancy between community delirium research and clinical practice, heterogeneity in diagnostic and screening tools, and a lack of data on dementia and delirium subtypes. Future research should address the heterogeneity between sample populations and determine the validity of delirium screening tools in people with dementia in the community.

## CONFLICTS OF INTEREST STATEMENT

The authors declare no conflicts of interest. Author disclosures are available in the Supporting Information.

## Supporting information




Supporting Information

Supporting Information

Supporting Information

Supporting Information

Supporting Information

Supporting Information

Supporting Information

Supporting Information
