# Supplementary material for: The prevalence and incidence of delirium superimposed on dementia in community settings: A systematic review and meta‐analysis
Source: Alzheimers Dement (Amst). 2026 Jun 18;18(2):e70398. doi: 10.1002/dad2.70398 (PMC13279347; doi:10.1002/dad2.70398)
Supplement: Supplementary file 2 — Supporting Information [file DAD2-18-e70398-s008.docx]

Appendix 2. Database searches

Medline

| **#** | **Query** | **Results from 6 Jan 2025** |
| --- | --- | --- |
| 1 | exp Dementia/ | 223,621 |
| 2 | Cognitive Dysfunction/ | 44,858 |
| 3 | dementia*.mp. [mp=title, book title, abstract, original title, name of substance word, subject heading word, floating sub-heading word, keyword heading word, organism supplementary concept word, protocol supplementary concept word, rare disease supplementary concept word, unique identifier, synonyms, population supplementary concept word, anatomy supplementary concept word] | 178,580 |
| 4 | Alzheimer*.mp. [mp=title, book title, abstract, original title, name of substance word, subject heading word, floating sub-heading word, keyword heading word, organism supplementary concept word, protocol supplementary concept word, rare disease supplementary concept word, unique identifier, synonyms, population supplementary concept word, anatomy supplementary concept word] | 225,777 |
| 5 | (cognitiv* adj4 declin*).mp. [mp=title, book title, abstract, original title, name of substance word, subject heading word, floating sub-heading word, keyword heading word, organism supplementary concept word, protocol supplementary concept word, rare disease supplementary concept word, unique identifier, synonyms, population supplementary concept word, anatomy supplementary concept word] | 45,222 |
| 6 | (cognitiv* adj4 impair*).mp. [mp=title, book title, abstract, original title, name of substance word, subject heading word, floating sub-heading word, keyword heading word, organism supplementary concept word, protocol supplementary concept word, rare disease supplementary concept word, unique identifier, synonyms, population supplementary concept word, anatomy supplementary concept word] | 124,084 |
| 7 | (lewy* adj2 bod*).mp. | 13,885 |
| 8 | 1 or 2 or 3 or 4 or 5 or 6 or 7 | 449,620 |
| 9 | exp Confusion/ | 18,804 |
| 10 | exp Consciousness Disorders/ | 48,739 |
| 11 | delirium.mp. [mp=title, book title, abstract, original title, name of substance word, subject heading word, floating sub-heading word, keyword heading word, organism supplementary concept word, protocol supplementary concept word, rare disease supplementary concept word, unique identifier, synonyms, population supplementary concept word, anatomy supplementary concept word] | 26,749 |
| 12 | "delirium superimposed on dementia".mp. [mp=title, book title, abstract, original title, name of substance word, subject heading word, floating sub-heading word, keyword heading word, organism supplementary concept word, protocol supplementary concept word, rare disease supplementary concept word, unique identifier, synonyms, population supplementary concept word, anatomy supplementary concept word] | 127 |
| 13 | comorbid delirium.mp. [mp=title, book title, abstract, original title, name of substance word, subject heading word, floating sub-heading word, keyword heading word, organism supplementary concept word, protocol supplementary concept word, rare disease supplementary concept word, unique identifier, synonyms, population supplementary concept word, anatomy supplementary concept word] | 12 |
| 14 | "acute confusion".mp. [mp=title, book title, abstract, original title, name of substance word, subject heading word, floating sub-heading word, keyword heading word, organism supplementary concept word, protocol supplementary concept word, rare disease supplementary concept word, unique identifier, synonyms, population supplementary concept word, anatomy supplementary concept word] | 456 |
| 15 | confusion* state.mp. [mp=title, book title, abstract, original title, name of substance word, subject heading word, floating sub-heading word, keyword heading word, organism supplementary concept word, protocol supplementary concept word, rare disease supplementary concept word, unique identifier, synonyms, population supplementary concept word, anatomy supplementary concept word] | 952 |
| 16 | exp Residence Characteristics/ | 82,576 |
| 17 | exp Nursing Care/ | 143,930 |
| 18 | exp Home Care Services/ | 52,366 |
| 19 | Long-Term Care/ | 29,389 |
| 20 | residence.mp. | 103,716 |
| 21 | residential facilities/ or assisted living facilities/ or group homes/ or halfway houses/ or homes for the aged/ | 24,135 |
| 22 | exp Primary Health Care/ | 202,863 |
| 23 | exp Housing for the Elderly/ | 1,664 |
| 24 | (community* or "care home*" or "nursing home" or "primary care" or "community care" or "home care" or "domiciliary care*" or "community-dwelling" or "living at home" or "living adj3 community" or "home").mp. | 1,206,261 |
| 25 | 16 or 17 or 18 or 19 or 20 or 21 or 22 or 23 or 24 | 1,558,710 |
| 26 | 9 or 10 or 11 or 12 or 13 or 14 or 15 | 80,357 |
| 27 | 8 and 25 and 26 | 1,191 |

Scopus:

dementia OR alzheimer OR "cognitive impairment" OR "cognitive decline"

AND

confusion OR "confusional state" OR delirium OR "delirium superimposed on dementia" OR "comorbid delirium" OR "acute confusion" OR "consciousness disorder"

AND

"community care" OR "home" OR "long term care" OR "care home" OR "nursing home" OR "primary care" OR "residential facilities" OR "domiciliary care" OR "community dwelling"

WOS

**((((ALL=(dementia)) OR ALL=(alzheimer)) OR ALL=(cognitive dysfunction)) OR ALL=(cognitive impairment)) OR ALL=(cognitive decline)**

AND

**((((ALL=(confusion)) OR ALL=(delirium)) OR ALL=(comorbid delirium)) OR ALL=(acute confusion)) OR ALL=(confusional state)**

AND

**(((((((((ALL=(community care)) OR ALL=(long term care)) OR ALL=(home care)) OR ALL=(community dwelling)) OR ALL=(housing for the aged)) OR ALL=(care home)) OR ALL=(nursing home)) OR ALL=(living at home)) OR ALL=(residential care)) OR ALL=(assisted living)**

Cochrane

dementia or alzheimer or "cognitive impairment" or "cognitive decline"

AND

confusion or delirium or "acute confusional state" or "delirium superimposed on dementia"

AND

community or long term care or "home care" or "community dwelling" or "nursing home" or "living at home" or "residential care" or "assisted living"

CINAHL

| **#** | **Query** | **Limiters/Expanders** | **Last Run Via** | **Results** |
| --- | --- | --- | --- | --- |
| S16 | S5 AND S9 AND S15 | Expanders - Apply equivalent subjects Search modes - Proximity | Interface - EBSCOhost Research Databases Search Screen - Advanced Search Database - CINAHL Plus | 779 |
| S15 | S10 OR S11 OR S12 OR S13 OR S14 | Expanders - Apply equivalent subjects Search modes - Proximity | Interface - EBSCOhost Research Databases Search Screen - Advanced Search Database - CINAHL Plus | 367,891 |
| S14 | homes for the aged/ or exp nursing homes/ or exp residential facilities/ or exp long-term care/ or aged specific care | Expanders - Apply equivalent subjects Search modes - Proximity | Interface - EBSCOhost Research Databases Search Screen - Advanced Search Database - CINAHL Plus | 5,051 |
| S13 | community-dwelling or community dwelling or living at home or community setting | Expanders - Apply equivalent subjects Search modes - Proximity | Interface - EBSCOhost Research Databases Search Screen - Advanced Search Database - CINAHL Plus | 48,217 |
| S12 | residential care or nursing home or long term care or care home | Expanders - Apply equivalent subjects Search modes - Proximity | Interface - EBSCOhost Research Databases Search Screen - Advanced Search Database - CINAHL Plus | 147,197 |
| S11 | home care services or home health care or home healthcare | Expanders - Apply equivalent subjects Search modes - Proximity | Interface - EBSCOhost Research Databases Search Screen - Advanced Search Database - CINAHL Plus | 34,346 |
| S10 | community care or community setting or community nurse or community nursing or primary care | Expanders - Apply equivalent subjects Search modes - Proximity | Interface - EBSCOhost Research Databases Search Screen - Advanced Search Database - CINAHL Plus | 212,669 |
| S9 | S6 OR S7 OR S8 | Expanders - Apply equivalent subjects Search modes - Proximity | Interface - EBSCOhost Research Databases Search Screen - Advanced Search Database - CINAHL Plus | 29,963 |
| S8 | consciousness disorders | Expanders - Apply equivalent subjects Search modes - Proximity | Interface - EBSCOhost Research Databases Search Screen - Advanced Search Database - CINAHL Plus | 1,717 |
| S7 | delirium or acute confusion or confusion or disorientation | Expanders - Apply equivalent subjects Search modes - Proximity | Interface - EBSCOhost Research Databases Search Screen - Advanced Search Database - CINAHL Plus | 28,333 |
| S6 | confusion | Expanders - Apply equivalent subjects Search modes - Proximity | Interface - EBSCOhost Research Databases Search Screen - Advanced Search Database - CINAHL Plus | 15,987 |
| S5 | S1 OR S2 OR S3 OR S4 | Expanders - Apply equivalent subjects Search modes - Proximity | Interface - EBSCOhost Research Databases Search Screen - Advanced Search Database - CINAHL Plus | 195,874 |
| S4 | cognitive decline or cognitive impairment or cognitive function | Search modes - Proximity | Interface - EBSCOhost Research Databases Search Screen - Advanced Search Database - CINAHL Plus | 62,901 |
| S3 | cognitive impairment or cognitive dysfunction or cognitively impaired | Search modes - Proximity | Interface - EBSCOhost Research Databases Search Screen - Advanced Search Database - CINAHL Plus | 42,450 |
| S2 | alzheimers | Search modes - Proximity | Interface - EBSCOhost Research Databases Search Screen - Advanced Search Database - CINAHL Plus | 53,700 |
| S1 | TX dementia |  |  |  |
