# Supplementary material for: The prevalence and incidence of delirium superimposed on dementia in community settings: A systematic review and meta‐analysis
Source: Alzheimers Dement (Amst). 2026 Jun 18;18(2):e70398. doi: 10.1002/dad2.70398 (PMC13279347; doi:10.1002/dad2.70398)
Supplement: Supplementary file 3 — Supporting Information [file DAD2-18-e70398-s004.docx]

Appendix 3 inclusion criteria

| **Table 1. Inclusion and exclusion criteria** | | |
| --- | --- | --- |
|  | ***Included*** | ***Excluded*** |
| ***Population*** | Clinical diagnosis of dementia (any type)  Studies including family carers of people with dementia (unpaid carer, friends and family). | Mixed population of people with and people without dementia, with no separate analysis on the people with dementia.  Diagnosis of mild cognitive impairment.  Patients who are terminally ill (other than dementia), or those with alcohol or substance-related delirium.  Paid carers |
|  | A clinical diagnosis of delirium – so have delirium superimposed on dementia (DSD) |  |
| ***Concept*** | Studies that report the prevalence, incidence or occurrence of delirium superimposed on dementia (DSD) in community settings. | Studies that do not report prevalence, incidence or occurrence. |
| ***Context*** | Primary and social care settings. |  |
|  | Community settings including participant’s home, outpatient/private clinic, day care centre, nursing home, residential care home, long-term care, memory clinics.  Any country / language. | Emergency department, hospital, hospices, psychiatric units, intensive care units.  Hospital discharge towards home or nursing home (post-acute settings).  Community acquired delirium but the study’s setting is based in a hospital. |
| ***Study Design or methodological factors*** | Prevalence data or sufficient raw data provided for prevalence to be calculated.  Observational studies including cohort, case-control and cross-sectional studies.  Interventional studies reporting baseline prevalence, risk factors, or impact of DSD. | Insufficient data for prevalence of DSD to be calculated.  Interventional studies that report DSD prevalence, risk factors, or impact after intervention.  Reviews, conference abstracts, qualitative studies, brief reports, letters to the editor, commentaries.  Case reports. |
