# Supplementary material for: The prevalence and incidence of delirium superimposed on dementia in community settings: A systematic review and meta‐analysis
Source: Alzheimers Dement (Amst). 2026 Jun 18;18(2):e70398. doi: 10.1002/dad2.70398 (PMC13279347; doi:10.1002/dad2.70398)
Supplement: Supplementary file 4 — Supporting Information [file DAD2-18-e70398-s006.docx]

| Data for extraction | Item | Author notes |
| --- | --- | --- |
| Study details | Authors  Year  Country  Language  Study aim  Study objectives  Methods |  |
| Participant details | Number of participants with dementia  Age of participants with dementia  Gender of participants with dementia  Place of residence for participants  Setting of study  Stage/type of dementia  How dementia was diagnosed |  |
| Prevalence | How delirium was diagnosed  Who diagnosed delirium  Number of participants with dementia with delirium (prevalence)  Number of participants with dementia with delirium (incidence) |  |
| Conclusions | Conclusions  Recommendations for practice  Recommendations for future research  Recommendations for policy  Strengths  Limitations |  |

Appendix 4. Data extraction form
