# Supplementary material for: The prevalence and incidence of delirium superimposed on dementia in community settings: A systematic review and meta‐analysis
Source: Alzheimers Dement (Amst). 2026 Jun 18;18(2):e70398. doi: 10.1002/dad2.70398 (PMC13279347; doi:10.1002/dad2.70398)
Supplement: Supplementary file 5 — Supporting Information [file DAD2-18-e70398-s002.docx]

Appendix 5. JBI checklists

| JBI Critical Appraisal checklist for prevalence data | | | | | | | | | | |  |
| --- | --- | --- | --- | --- | --- | --- | --- | --- | --- | --- | --- |
| Study | Q1  Sample frame appropriate? | Q2  Were participants sampled appropriately? | Q3  Sample size adequate? | Q4  Subjects and the setting described in detail? | Q5  Data analysis with sufficient coverage of the sample? | Q6  Validity of methods | Q7  Condition measured in a standard, reliable way? | Q8  Appropriate statistical analysis? | Q9  Response rate adequate, or managed appropriately? | **Areas of potential risk of bias** |  |
| **Boorsma et al., 2012** | Yes | Unclear | Yes | Yes | Yes | Yes | Yes | Yes | Yes | **1/9** |  |
| **Morichi et al., 2018** | Yes | Yes | Yes | Yes | Yes | Yes | Yes | Yes | No | **1/9** |  |
| **Sandberg et al., 1998** | Yes | Yes | Yes | Yes | Yes | Yes | Yes | Yes | No | **1/9** |  |
| **Tremolizzo et al., 2021** | No | No | No | Yes | Yes | Yes | Yes | Yes | No | **4/9** |  |
| **Quispel-Aggenbach et al., 2019** | Yes | Yes | Yes | Yes | Yes | Yes | Yes | Yes | No | **1/9** |  |
| **Manni et al., 2021** | Yes | Yes | Yes | Yes | Yes | Yes | Yes | Yes | No | **1/9** |  |
| **McCusker et al., 2011** | Yes | Unclear | Yes | Yes | Yes | Yes | Yes | Yes | Yes | **1/9** |  |

| JBI Critical Appraisal checklist for cross sectional data | | | | | | | | | |
| --- | --- | --- | --- | --- | --- | --- | --- | --- | --- |
| Study | Q1  Inclusion criteria clearly defined? | Q2  Study subjects and setting described in detail? | Q3  Was the exposure measured in a valid and reliable way? | Q4  Objective, standard criteria used for measurement of condition? | Q5  Were confounding factors identified? | Q6  Were strategies to deal with confounding factors stated? | Q7  Were the outcomes measured in a valid and reliable way? | Q8  Was appropriate statistical analysis used? | **Areas of potential risk of bias** |
| **Oudewortel et al., 2021** | No | Yes | Yes | Yes | Yes | Yes | Yes | Yes | **1/8** |
| **Mentes et al., 1999** | No | No | Unclear | Unclear | Yes | Yes | Unclear | Yes | **5/8** |
| **Zazzara et al., 2021** | No | Yes | Yes | Yes | Yes | Yes | Yes | Yes | **1/8** |
| **Mathillas et al. 2013** | No | Yes | Yes | Yes | Yes | Yes | Yes | Yes | **1/8** |

| JBI Critical Appraisal checklist for cohort data | | | | | | | | | | | | |
| --- | --- | --- | --- | --- | --- | --- | --- | --- | --- | --- | --- | --- |
| Study | Q1  Group similarity | Q2  Were the exposures measured similarly to assign people to groups? | Q3  Was the exposure measured in a valid and reliable way? | Q4  Confounding factors identified? | Q5  Strategies to deal with confounding factors stated? | Q6  Were the groups/participants free of the outcome? | Q7  Were the outcomes measured in a valid and reliable way? | Q8  Was the follow up time reported and sufficient to be long enough? | Q9  Follow-up complete or were reasons to loss to follow up described? | Q10  Strategies for incomplete follow up utilised? | Q11  Appropriate statistical analysis used? | **Areas of potential risk of bias** |
| **Holmes et al. 2011** | Yes | Yes | Yes | Yes | Yes | Yes | Yes | Yes | Yes | Unclear | Yes | **1/11** |
| **Skretteberg et al.,** | Yes | Yes | Yes | Unclear | Yes | Yes | Yes | Yes | Unclear | No | Yes | **3/11** |
| **Katipoglui et al., 2022** | Yes | Yes | Yes | Yes | Yes | N/A | Yes | Yes | Unclear | No | Yes | **2/11** |
| **Delgado et al., 2020** | N/A | Yes | Yes | No | No | N/A | Yes | Yes | Yes | N/A | Yes | **2/11** |
| **Katipoglu et al., 2023** | Yes | Yes | Yes | Yes | Yes | Yes | Yes | Yes | Unclear | Unclear | Yes | **2/11** |
| **Vida et al., 2006** | Yes | Yes | Yes | Yes | Yes | N/A | Yes | Yes | Yes | No | Yes | **1/11** |
| **Cole et al., 2011** | Yes | Yes | Yes | Yes | Yes | Yes | Yes | Yes | Yes | Unclear | Yes | **1/11** |
| **Erikson et al., 2007** | Yes | Yes | Unclear | Yes | Yes | N/A | Yes | Yes | Yes | Unclear | Yes | **2/11** |
| **Hasegawa et al., 2013** | Yes | Yes | Yes | No | No | N/A | Yes | N/A | N/A | N/A | Yes | **2/11** |

| JBI Critical Appraisal checklist for RCT data | | | | | | | | | | | | | | | |
| --- | --- | --- | --- | --- | --- | --- | --- | --- | --- | --- | --- | --- | --- | --- | --- |
| Study | Q1  True randomization used? | Q2  Allocation concealed? | Q3  Treatment groups similar at the baseline? | Q4  Blinded participants? | Q5  Blinded treatment? | Q6  Treatment groups treated similar? | Q7  Outcome assessors blinded? | Q8  Outcomes measured in the same way? | Q9  Outcomes measured reliably? | Q10  Follow up complete or differences adequately described and analysed? | Q11  Participants analysed in groups which they were randomized? | Q12  Appropriate statistical analysis used? | Q13  Trial design appropriate and deviations from the standard design accounted for? | **Areas of potential risk of bias** |  |
| **Bogarets et al. 2024** | Yes | Yes | Yes | No | Yes | Yes | Yes | Yes | Yes | Yes | Yes | Yes | Yes | **1/11** |  |
| **Dyer et al., 2020a** | Yes | Yes | Yes | Yes | Yes | Yes | Yes | Yes | Yes | Yes | Yes | Yes | Yes | **0/11** |  |
| **Dyer et al., 2020b** | Yes | Yes | Yes | Yes | Yes | Yes | Yes | Yes | Yes | Yes | Yes | Yes | Yes | **0/11** |  |
| **Santana et al., 2021** | Yes | N/A | Yes | No | Yes | Yes | Yes | Yes | Yes | Yes | Yes | Yes | Yes | **1/11** |  |

| JBI Critical Appraisal checklist for case control data | | | | | | | | | | | | |  |
| --- | --- | --- | --- | --- | --- | --- | --- | --- | --- | --- | --- | --- | --- |
| Study | Q1  Groups comparable other than the presence or absence of disease? | Q2  Cases and controls matched appropriately? | Q3  Same criteria used for identification of cases and controls? | Q4  Exposure measured in a standard, valid and reliable way? | Q5  Exposure measured in the same? | Q6  Confounding factors identified? | Q7  Strategies to deal with confounding factors stated? | Q8  Outcomes assessed in a standard, valid and reliable way? | Q9  Exposure period of interest long enough to be meaningful? | Q10  Appropriate statistical analysis used? | **Score** |  |  |
| **Stroomer-van Wijk et al., 2016** | Yes | No | Unclear | Yes | Yes | Yes | Yes | Yes | Yes | Yes | **2/10** |  |  |
| **Fick et al., 2005** | Yes | Yes | Yes | Yes | Yes | No | No | Yes | Yes | Yes | **2/11** |  | |
| **Lerner et al., 1999** | Yes | Yes | Yes | Yes | Yes | No | No | Yes | Yes | Yes | **2/11** |  | |
