# Supplementary material for: The prevalence and incidence of delirium superimposed on dementia in community settings: A systematic review and meta‐analysis
Source: Alzheimers Dement (Amst). 2026 Jun 18;18(2):e70398. doi: 10.1002/dad2.70398 (PMC13279347; doi:10.1002/dad2.70398)
Supplement: Supplementary file 6 — Supporting Information [file DAD2-18-e70398-s005.docx]

Appendix 6. Study Characteristics

| Appendix 6. Study characteristics | | | | | | | | | |
| --- | --- | --- | --- | --- | --- | --- | --- | --- | --- |
| **Study** | **Study location** | **Study aim** | **Study design** | **Study setting** | **Place of residence for participants** | **Number of participants with dementia** | **Age in years** | **Gender**  **(Female)** | **Dementia subtype and severity (mean and SD unless reported otherwise)** |
| Bogaerts et al., (2024) | The Netherlands | To assess if discontinuing antihypertensive treatment reduces neuropsychiatric symptoms and maintains quality of life in people with dementia. | Randomised controlled trial | Nursing home | Nursing homes | 205 | 85.95 (IQR 79.7-89.1) | 163 (80%) | AD: n=82 (40%)  VD: n=32 (16%)  FTD: 4 (2%)  LBD: n=2 (0.5%)  Mixed or other: n= 39 (19%)  Unknown: n=47 (23%)  Moderate to severe dementia.  Reisberg GDS Scale 5: n=86 (42%)  Reisberg GDS Scale 6: n=102 (50%)  Reisberg GDS Scale 7: n=17 (n=8%) |
| Boorsma et al., (2012) | The Netherlands | To compare the prevalence, incidence and risk factors of delirium in nursing and residential homes. | Retrospective cohort study | Nursing home and residential homes | Nursing home and residential homes | 771 | Not reported | Not reported | Not reported |
| Cole et al., (2011) | Canada | To determine the incidence of and risk factors of subsyndromal delirium in older people in long-term care. | Prospective cohort study | Long-term care | Long-term care | 49 | ≥65 years | Not reported | AD: n = 14 (14%)  VD: n = 11 (11%)  Others and not specified: n = 17 (16%)  Mixed: n = 7 (7%)  Severity not reported. |
| Delgado et al., (2022) | The UK | To estimate the association between higher continuity of GP care and prescribing and the impact on health outcomes in people with dementia. | Retrospective cohort study | General practice | Community dwelling | 9324 | 84.5 (SD 7.4) | 6124 (66%) | Not reported subtypes but YOD or rare forms of dementia (CJD FTD, Huntington’s disease) were excluded.  Severity not reported. |
| Dyer et al., (2020a) | Ireland | To evaluate the association between benzodiazepines and cognitive decline in people with Alzheimer’s. | Randomised controlled trial | Community dwelling | Community dwelling | 448 | 72.46 (SD 8.2) | 279 (63%) | “Mild to moderate” AD.  Baseline ADAS-Cog: 33.95 (10.55) |
| Dyer et al., 2020b | Ireland | To assess the prevalence of sedative medication use and the long-term association with adverse events | A secondary analysis of a randomised controlled trial (Dyer et al., 2020a) | Community dwelling | Community dwelling | 510 | 72.8 (SD 8.3) | (Raw score not reported) 62% | “Mild to moderate” AD.  Baseline ADAS-Cog: 35.14 (10.51)  Baseline DAD: 28.61(1.48) |
| Erikson et al., (2008) | Sweden | To identify predisposing risk factors for falls in older people with and without dementia. | Prospective cohort study | Residential care facilities | Residential care facilities | 103 | 83.6 (SD 6.3) | 83 (80%) | Not reported subtypes.  Baseline MMSE: 12.2 (7.1) |
| Fick et al., (2005) | The USA | To examine the 3-year occurrence, health care utilisation and costs associated with DSD. | Case-control study | Community dwelling | Community dwelling | 7347 | 75.15 (SD 5.0) | 839 (60%) | Not reported |
| Hasegawa et al., (2013) | Japan | To investigate the frequency of DSD in each dementia subtype & the association of delirium and cerebrovascular disease. | Prospective cohort study | Outpatients in memory clinic of psychiatric hospital | Community outpatients | 206 | 81.4 (SD 6.0) | 142 (69%) | AD: n=129 (63%)  VD: n=32 (16%)  DLB: n=22 (11%)  FTLD: n=4 (0.2%)  'other’: n=18 (1%)  Baseline MMSE: 14.7 (6.1) |
| Holmes et al., (2011) | The UK | To describe the mortality risk of older people with delirium. | Prospective cohort study | Community dwelling | Community dwelling | 300 | 82.80 (SE 0.4) | 198 (56%) | “Mild to severe” AD. |
| Katipoglu et al., (2022) | Turkey | To assess the prevalence of delirium in patients with dementia and the association between inflammatory markers and mortality in DSD. | Retrospective cohort study | Tertiary outpatient geriatric clinic | Community dwelling | 615 | 78.1 (SD 6.8) | 402 (65%) | Not reported subtypes.  “Moderate to severe dementia”  Baseline MMSE: 18.0 (6.4) |
| Katipoglu et al., (2023) | Turkey | To understand the association between DBI and DSD. | Retrospective cohort study | Nursing homes | Nursing homes | 721 | 88.4 (SD 6.7) | 466 (65%) | AD: (n= 513, 71%)  VD: (n=148, 21%)  FTD: (n=10, 1%)  Mixed and other dementia: (n=50, 7%).  Baseline CDR: 1.8 (0.7) |
| Lerner et al., (1997) | The USA | To examine the incidence and circumstances of delirium in people with AD. | Retrospective case control study | AD research registry at Case Western Reserve University | Community dwelling | 199 | 75.9 (SD 7.3) | Not reported | AD  Not reported severity. |
| Manni et al., (2021) | Italy | To measure the prevalence of delirium, the clinical features, and outcomes in people with dementia referred to a memory clinic. | Retrospective cohort study | Memory clinic | Lived at home | 109 | 84. (SD 7.74) | 65 (60%) | VD: n= 38 (35%)  AD: n=28 (26%)  VD & AD: n= 14 (13%)  FTD: n= 3 (3%)  PDD: n= 1 (1%)  LWD' n= 5 (5%)  MCI: n= 15 (14%)  Other: n= 5 (5%)  Baseline CIRS severity index: 2.51 (1.15) |
| Mathillias et al., (2013) | Sweden | To investigate the 30 - day prevalence of delirium in “very old people”. | Cross-sectional study | Community | In their own homes, independent housing or institutional living | 299 | 91.5 (SD 4.7) | 139 (46%) | Not reported subtypes.  Baseline MMSE: 12.4 (7.5) |
| McCusker et al., (2011) | Canada | To describe the prevalence and 6-month incidence of delirium in people who reside in long-term care facilities. | Prospective cohort study | Long-term care facilities | Long-term care facilities | MMSE score ≤10:  With dementia: n= 51  Without dementia: n=4  Total (n=55) | Over 65 | Not reported | AD: n=25 (46%)  VD: n=3 (6%)  Mixed: n=4 (7%)  Others & not specific: n=19 (36%)  Baseline MMSE: score of 10 or less. |
| Mentes et al., (1999) | The USA | To validate whether a conceptual model which was developed from acute care findings, could identify acute confusion risk variables. | Cross-sectional study | Nursing homes | Nursing homes | 1027 | Not reported | Not reported | Not reported |
| Morichi et al., (2018) | Italy | To collect data about delirium and its associated factors in people aged 65 and over. | Cross-sectional study | Nursing homes | Nursing homes | 754 | Not reported | Not reported | Not reported |
| Oudewortel et al., 2021 | The Netherlands | To investigate the association of anticholinergic drugs with the prevalence of delirium. | Cross-sectional study | Nursing homes | Nursing homes | 2108 | <75 years: n=287 (14%)  75-84 years: n=718 (34%)  75-84 years: n=718 (34%)  85+: n=1103 (52%) | 1592 (76%) | Not reported subtypes.  Baseline CPS: Mild (0-1): n= 180 (9%)  Moderate (2-4): n=921 (44%)  Severe (5-6): n= 968 (46%)  Missing: n= 39 (2%) |
| Quispel-Aggenbach et al., (2019) | The Netherlands | To investigate the prevalence and risk factors of delirium in older people | Cross-sectional study | At the patients (care) home | Living at home or in care centres. | 34 | Not reported | Not reported | Not reported |
| Sandberg et al., (1998) | Sweden | To examine the prevalence of dementia, delirium and psychiatric symptoms among “the elderly”. | Cross-sectional study | Nursing homes, “old people’s home”, home medical care | Nursing homes, “old people’s home”, home medical care | Not reported | Age 75+ | Not reported | Not reported |
| Santagata et al., (2021) | Italy | To evaluate the effect of doll therapy in the management of BPSD, reduction of carer burden and incidence of delirium. | Randomised controlled trial | Nursing homes | Nursing homes | 52 | 86.6 (SE 6.5) | 83% | Not reported subtypes.  “Moderate to severe dementia”  Baseline CDR: 2.55 (SE 0.65) |
| Skretteberg et al., (2022) | Norway | To identify how often delirium occurred during acute events, the risk factors and course of delirium in nursing home patients. | Prospective cohort study | Nursing homes | Nursing homes | 116 | Not reported | Not reported | AD: n=29 (20%)  VD: n=13 (9%) Unspecified: n=70 (48%)  Others: n=4 (3%)  Not reported severity. |
| Stroomer-van Wijk et al., (2016) | The Netherlands | To study delirium symptoms and underlying somatic disorders in “elderly outpatients”. | Case control study | Outpatient old age psychiatry department | Living at home or in a ‘home for the elderly’ | 68 | 86.15 (SD 6.96) | 51 (75%) | Not reported subtypes.  Delirium+ dementia group MMSE baseline: 18.2 (5.8)  Dementia only group MMSE baseline: 20.1 (5.4) |
| Tremolizzo et al., (2021) | Italy | To assess the prevalence of delirium in older people living at home. | Prospective cohort study | The participant’s home | Living at home | 35 | Not reported | Not reported | Not reported |
| Vida et al., (2006) | Canada | To compare differences in ADL in patients diagnosed with delirium in the emergency department with non-delirious patients. | Prospective cohort study | Examined in emergency department | Lived at home | 132 | 82.6 (SD not reported) | 80 (60%) | Not reported subtypes.  Baseline MMSE: 10.25 (SD not reported)  Baseline IQCODE: 4.25 (SD not reported) |
| Zazzara et al., (2022) | Italy | To identify different comorbidity patterns and understand the clinical, functional and behavioural phenotypes in nursing home residents with dementia. | Cross-sectional study | Nursing home | Nursing home | 2563 | <70: n=137 (6%)  70-79: n=372 (15%)  80-89: n=1233 (48%)  ≥90: n=821 (32%) | 1877 (73%) | AD: (n=634, 24.7%). Does not report other subtypes.  CPS scores:  Moderate impairment: n=863 (33.7%)  Severe impairment: n=1426 (55.6%) |
| AD: Alzheimer’s disease; ADAS-Cog; Alzheimer’s Disease Assessment Scale-Cognitive Subscale; ADL: activities of daily living; CDR: Clinical Dementia Rating; CJD: Creutzfeldt-Jakob disease; CPS: Cognitive Performance Scale; DAD: Disability Assessment for Dementia; DSD: delirium superimposed on dementia; FTD: fronto-temporal dementia; GDS: Global Deterioration Scale; IQCODE: Informant Questionnaire on Cognitive Decline in the Elderly; IQR: interquartile range; LWD: lewy body dementia; MCI: mild cognitive impairment; MMSE: Mini-Mental State Examination; RCT: randomised controlled trial; SD: standard deviation; SE: standard error; VD: vascular dementia; YOD: young onset dementia | | | | | | | | | |
