# Supplementary material for: The prevalence and incidence of delirium superimposed on dementia in community settings: A systematic review and meta‐analysis
Source: Alzheimers Dement (Amst). 2026 Jun 18;18(2):e70398. doi: 10.1002/dad2.70398 (PMC13279347; doi:10.1002/dad2.70398)

Appendix 7 – meta analyses and funnel plots

7a – Funnel plot for Figure 2, total pooled prevalence


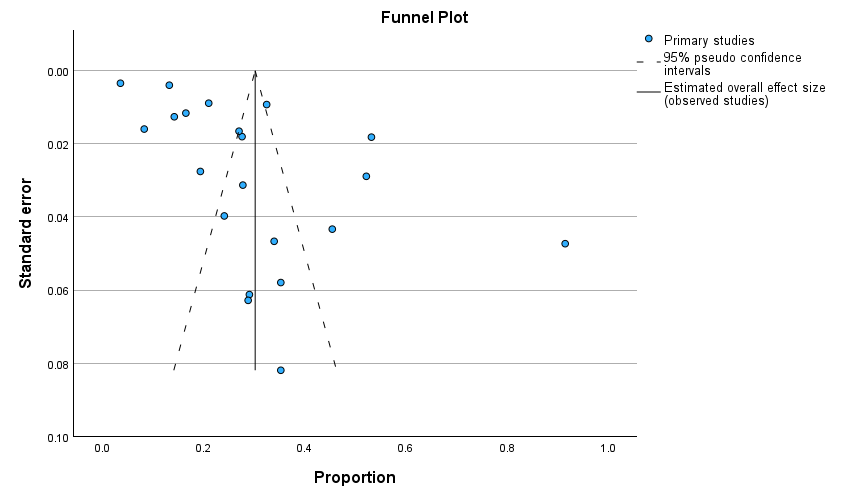


Appendix 7b – sensitivity analysis pooled prevalence excluding papers with 4 or more areas of potential risk of bias


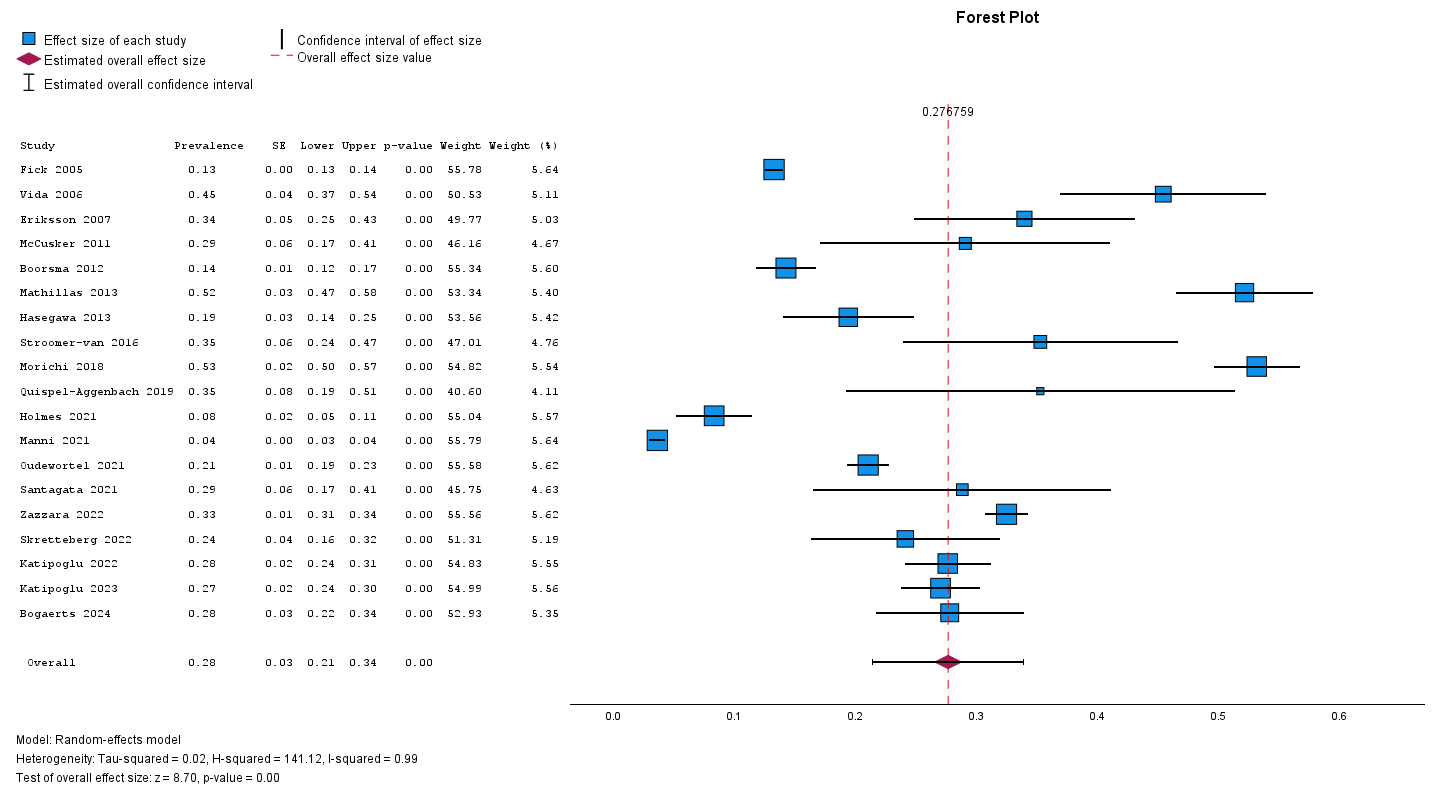


7c forest plot of sensitivity analysis total pooled prevalence excluding papers with 4 or more areas of potential risk of bias


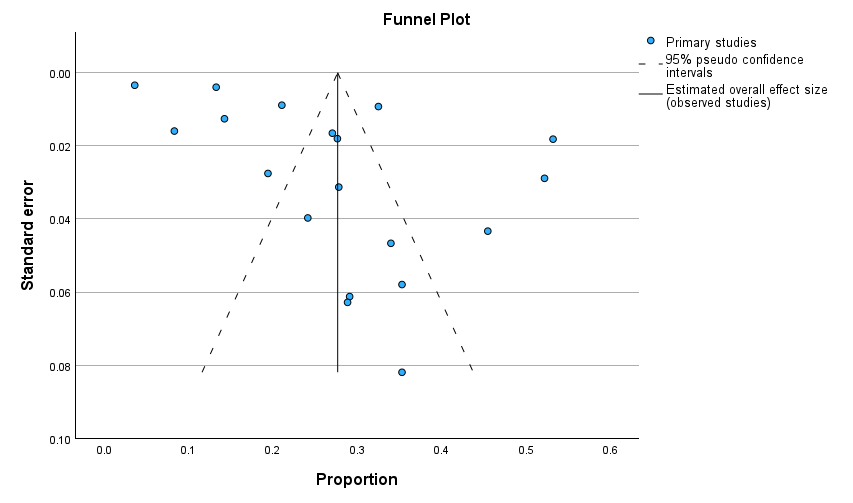


Appendix 7d - country subgroup analysis


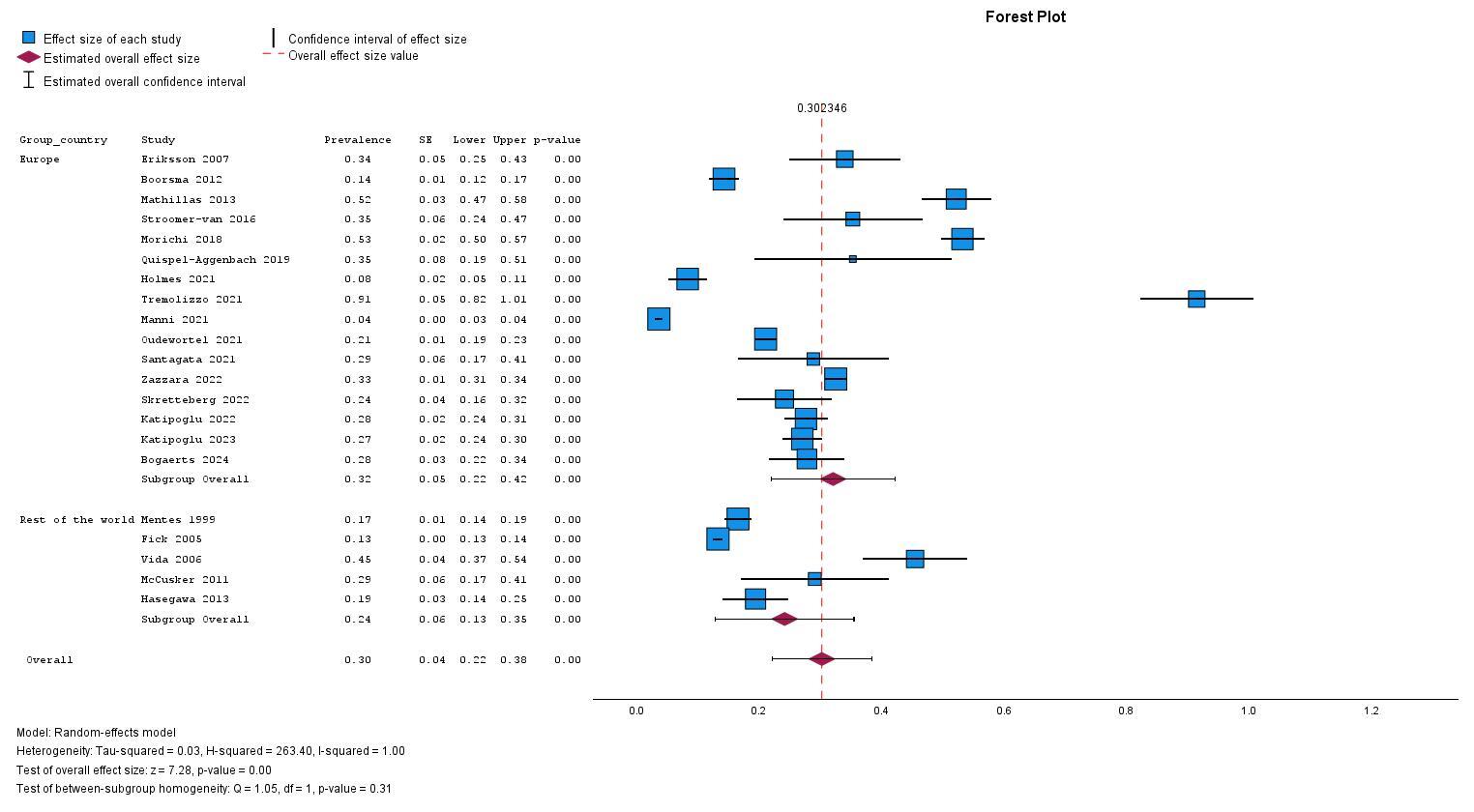


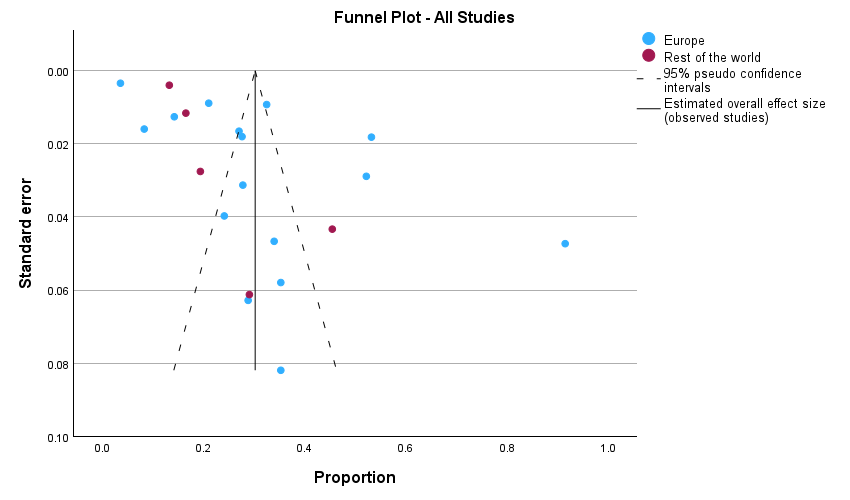


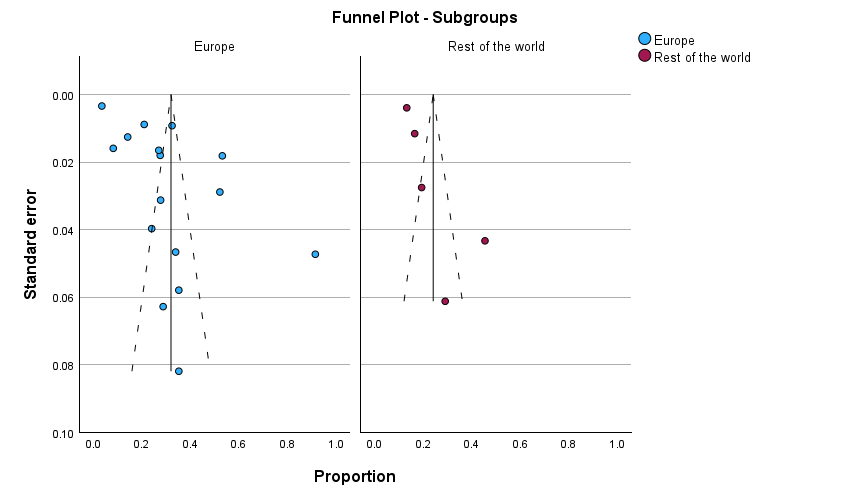


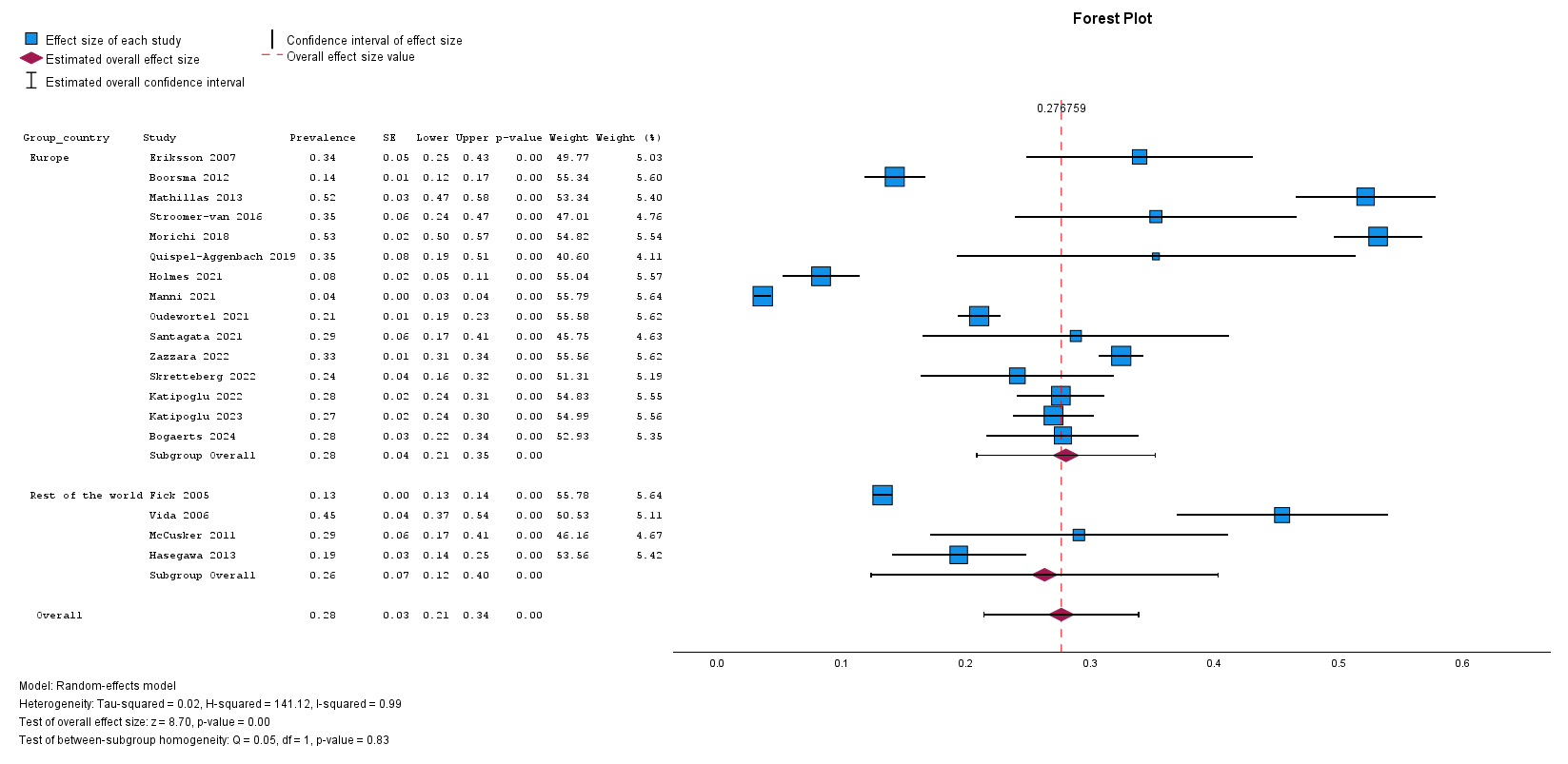
Appendix7 e – subgroup analysis of country excluding papers with 4 or more areas of potential risk of bias

Appendix 7f – subgroup analysis of setting


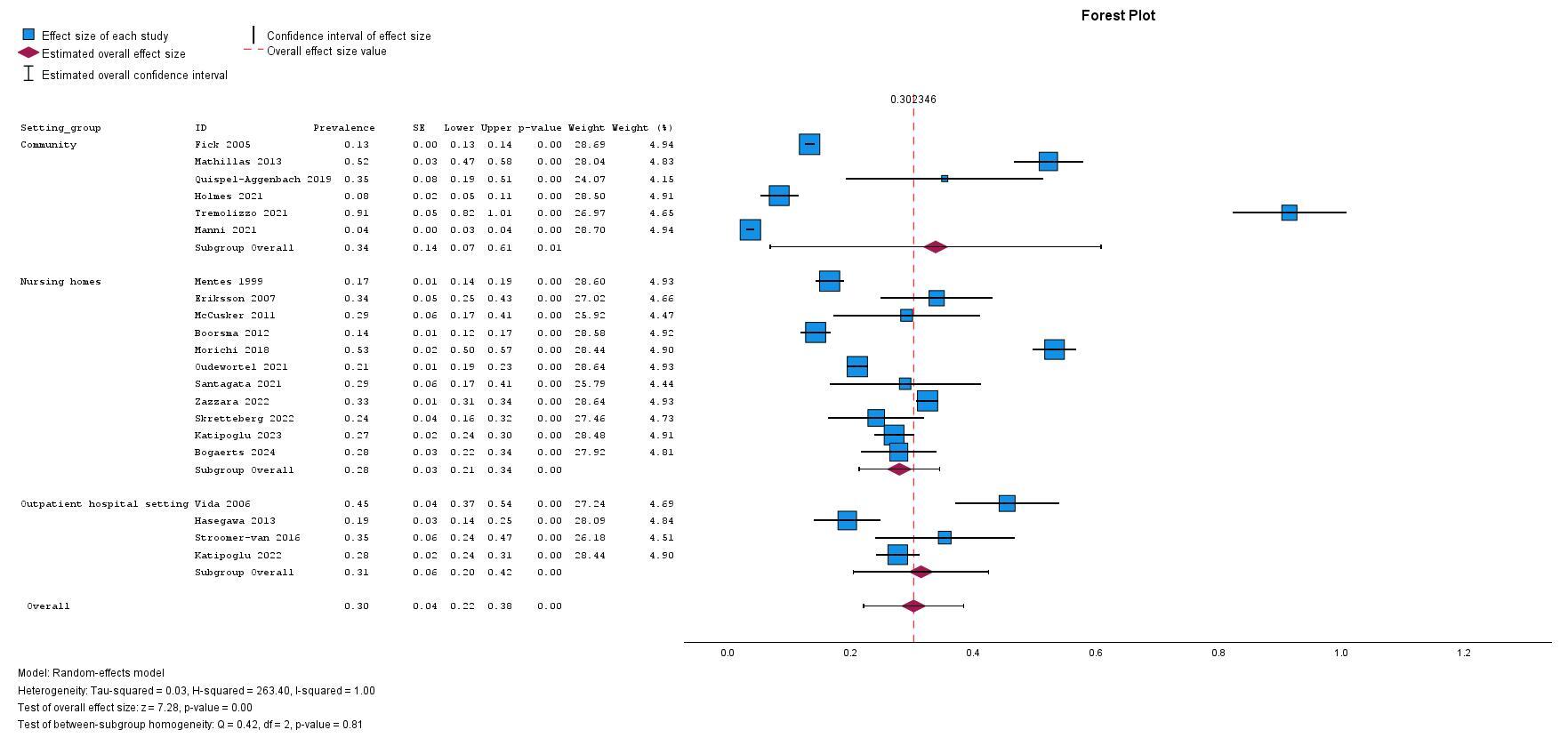


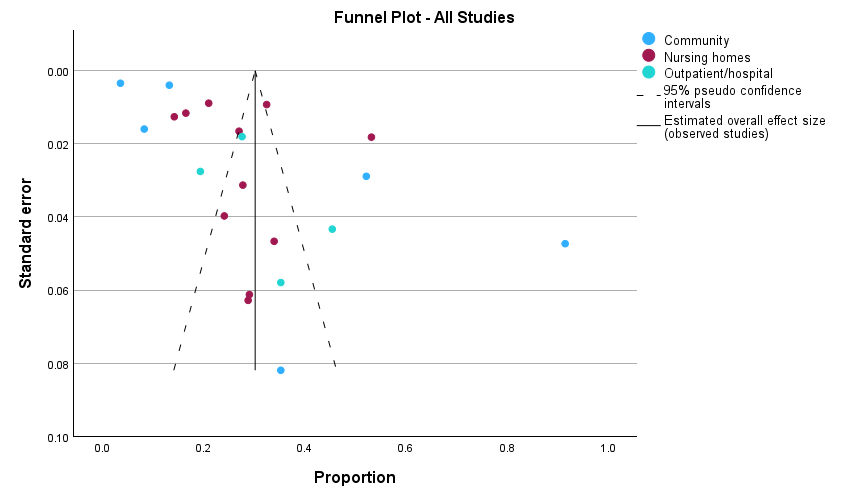


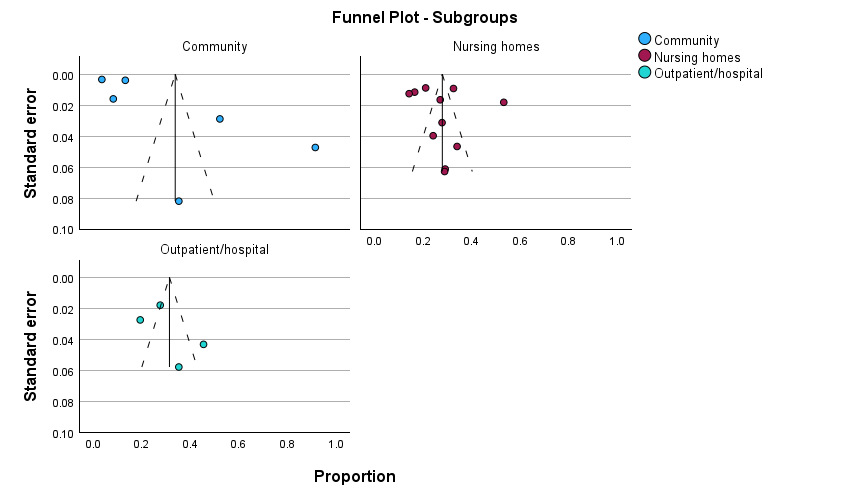


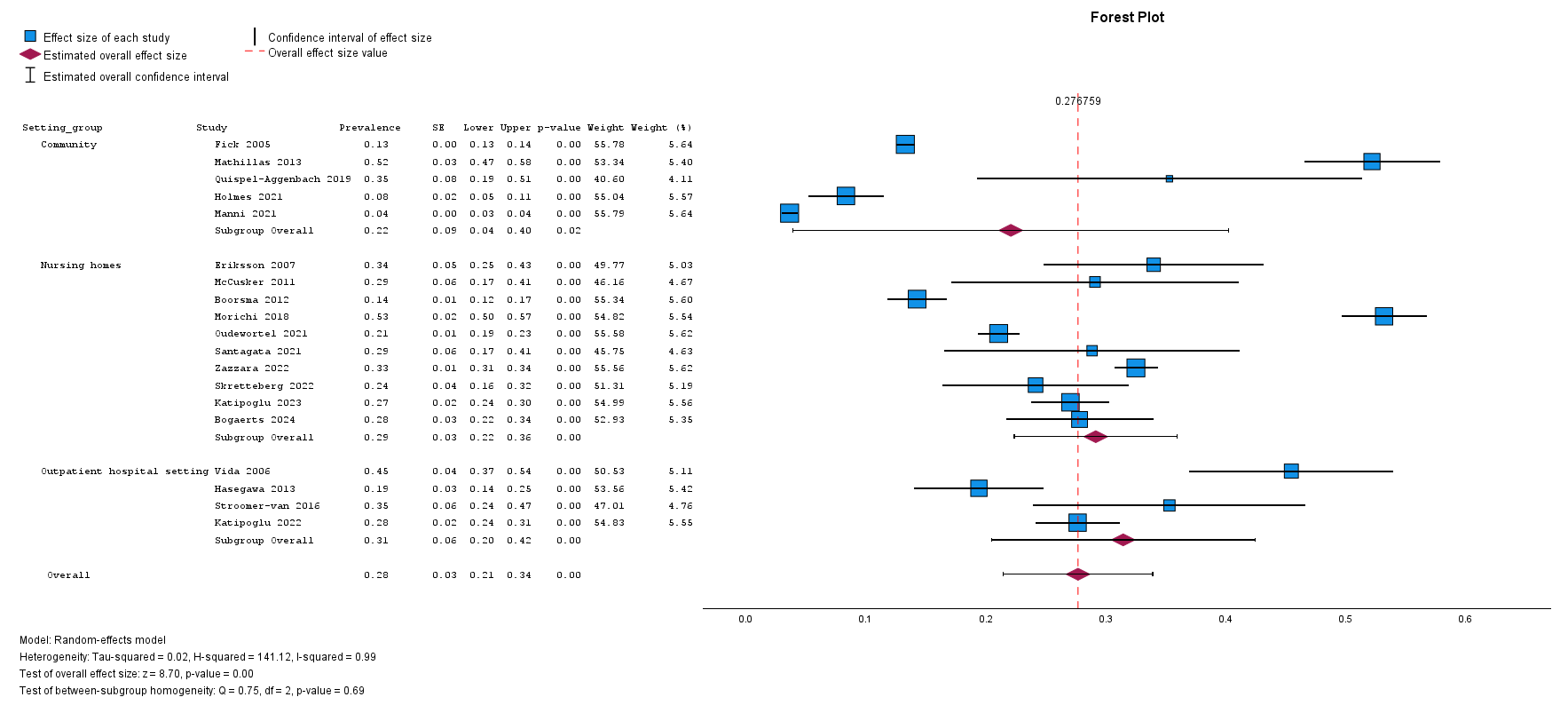
Appendix 7g –subgroup analysis of setting excluding papers with 4 or more areas of potential risk of bias


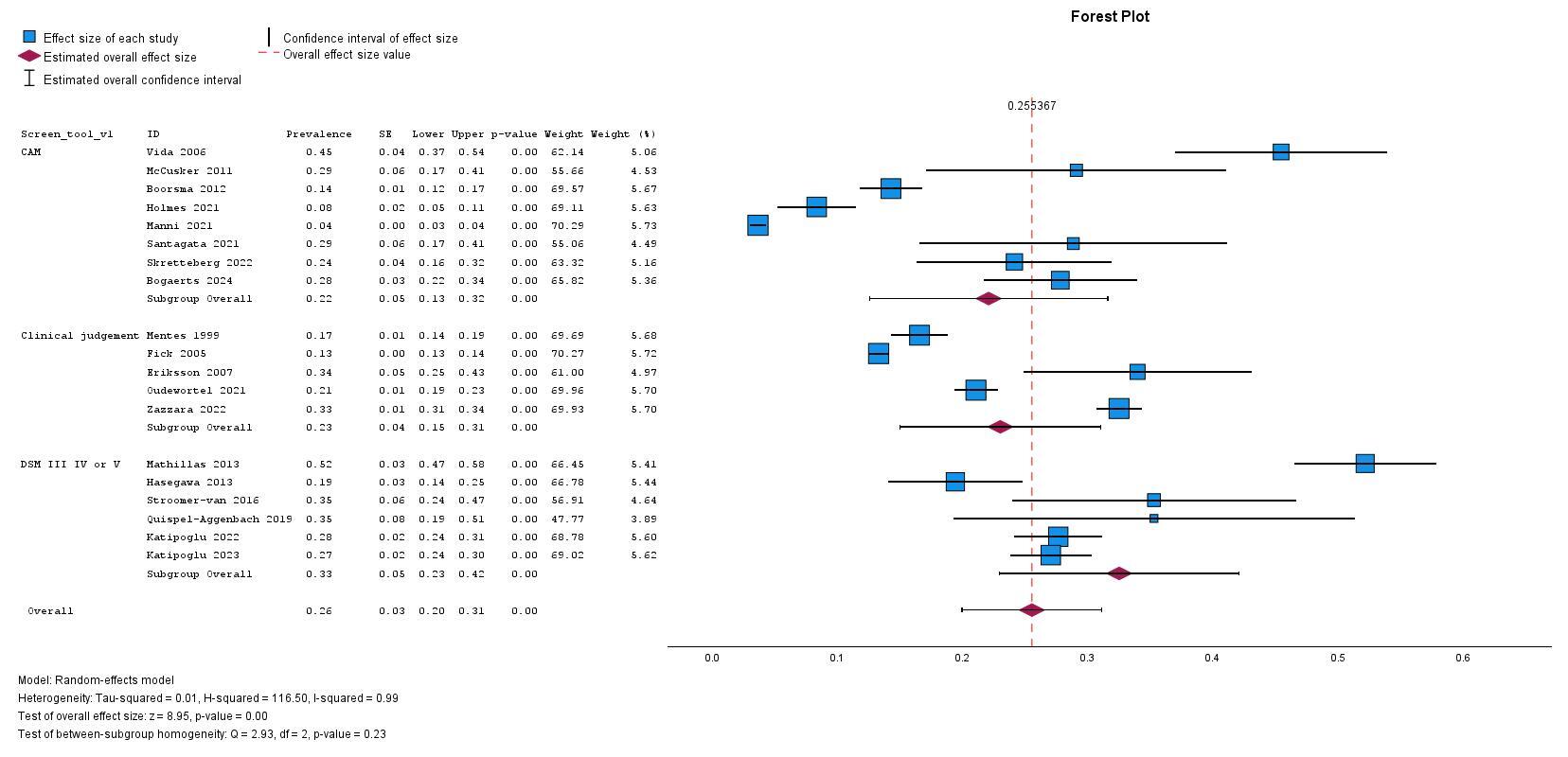
Appendix 7h –subgroup analysis of screening/diagnostic tool


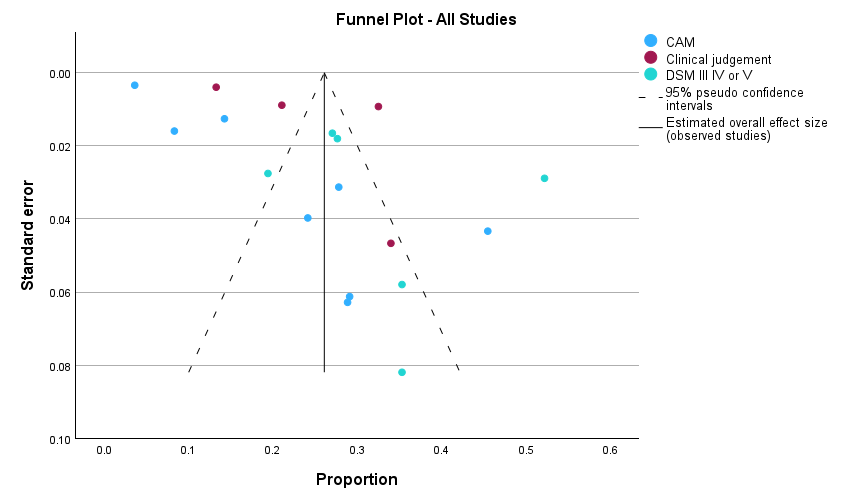


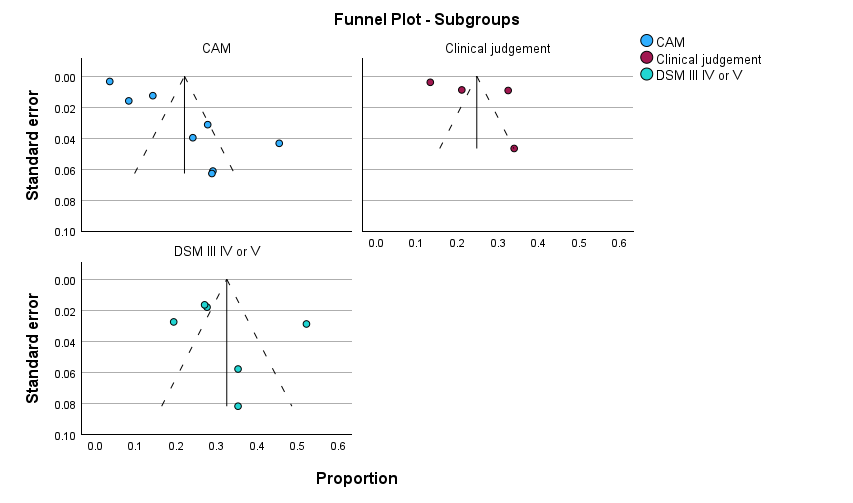

Supplement: Supplementary file 7 — Supporting Information [file DAD2-18-e70398-s001.docx]
